# New Insights Regarding Genetic Aspects of Childhood Obesity: A Minireview

**DOI:** 10.3389/fped.2018.00271

**Published:** 2018-10-04

**Authors:** Cristina Oana Mǎrginean, Claudiu Mǎrginean, Lorena Elena Meliţ

**Affiliations:** ^1^Department of Pediatrics, University of Medicine and Pharmacy Târgu Mureş, Târgu Mureş, Romania; ^2^Department of Obstetrics and Gynecology, University of Medicine and Pharmacy Târgu Mureş, Târgu Mureş, Romania

**Keywords:** obesity, genetics, children, etiology, dietary interventions

## Abstract

**Introduction:** Childhood obesity is occurring at alarming rates in both developed and developing countries. “Obesogenic” environmental factors must be associated with variants of different risk alleles to determine polygenic or common obesity, and their impact depends on different developmental stages.The interaction between obesogenic environment and genetic susceptibility results in the so-called polygenic forms of obesity. In contrast, monogenic and syndromic obesity are not influenced by environmental events. Therefore, this review aimed to underline the roles of some of the most studied genes in the development of monogenic and polygenic obesity in children.

**Results:** Among the most common obesity related genes, we chose the fat mass and obesity-associated (FTO) gene, leptin gene and its receptor, tumor necrosis factor alpha (TNF-α), the melanocortin 4 receptor gene (MC4R), Ectoenzyme nucleotide pyrophosphate phosphodiesterase 1 (ENPP1), and others, such as peroxisome proliferator-activated receptor gamma (PPARG), angiotensin-converting enzyme (ACE), glutathione S-transferase (GST), and interleukin-6 (IL-6) genes. The roles of these genes are complex and interdependent, being linked to different cornerstones in obesity development, such as appetite behavior, control of food intake and energy balance, insulin signaling, lipid and glucose metabolism, metabolic disorders, adipocyte differentiation, and so on.

**Conclusions:** Genetic predisposition is mandatory, but not enough to trigger obesity.Dietary interventions and proper lifestyle changes can prevent obesity development in genetically predisposed children. Further studies are needed to identify the precise role of both genetic and obesogenic factors in the development of childhood obesity in order to design effective preventive methods.

## Summary

Childhood obesity has become recently a global epidemic due to its high rates in both developed and developing countries. In Romania, a recent study found that one in four children is overweight or obese, and identified male gender, prepuberal age, and urban environment as risk factors for overweight. This major public health problem is a result of the interaction between environmental factors and individual genetic susceptibility. “Obesogenic” environmental factors must be associated with variants of different risk alleles to determine polygenic or common obesity, and their impact depends on different developmental stages.

## Introduction

Recently, childhood obesity has become a global epidemic, occurring at high rates in both developed and developing countries (1). Most studies regarding this health burden were performed on adult populations, and only a few of them included children. In Romania, a recent study found that one in four children is overweight or obese and identified male sex, prepubertal age, and urban environment as risk factors for incidences of overweight (2). This major public health problem is a result of the interaction between environmental factors and individual genetic susceptibility. Heritability plays a key role in the development of childhood obesity, with a rate of determinism as high as 70%, and it was proven to be higher in children than adults (3, 4). Obesogenic environment is only a trigger, and not the leading-cause for excessive weight gain, a genetic susceptibility to fat gain being mandatory for an individual to become obese (5–7). Therefore, environmental factors just favor the phenotypic expression in individuals programmed to become obese (8). Moreover, the impact of obesogenic environment differs during different developmental stages. For example, Silvenstoinen et al. performed a systematic review on twins and adopted children and proved that environmental factors moderately influence BMI variation only up to the age of 13 years, their effect disappearing beyond this age (6). It is true BMI curve in children is therefore genetically programmed, but at the same time environmental circumstances are able to modify this curve (8). Another well-documented fact is that fetal and early postnatal environmental events, such as maternal nutritional status during pregnancy, maternal smoking, gestational diabetes, increased birth weight, rapid weight gain, or feeding practices, may also influence the development of obesity later in life (8–16). In addition, the expression of genetic risks predominates during infancy and early childhood leading to an earlier adiposity rebound and higher BMI in children that carry a genetic susceptibility (17–19).

Therefore, it is apparent that “obesogenic” environmental factors alone are not responsible for the development of obesity; they must be associated with variants of different risk alleles to determine polygenic or common obesity (20). Many studies focused on assessing the role of different genes in the determination of obesity and found that certain variants are associated with weight regulation and adipose tissue accumulation (21). Therefore, recent genome-wide association studies identified over 50 genetic loci that can be associated with obesity (22), resulting in polygenic forms of obesity. Polygenic' variants are defined as any of a group of alleles at different gene loci that express a combined effect on controlling the inheritance of a quantitative phenotype or which can modify the expression of a qualitative character. Therefore, certain traits can be the result of the simultaneous presence of DNA changes in multiple genes. In case of quantitative traits, it was proved that each allele owns a small effect and these effects can be additive or non-additive. It is generally assumed that many of these polygenic variants are involved in body weight regulation and in individuals who harbor multiple of these variants, obesity can occur. Thus, the hypothesis of polygenic obesity implies that every obese individual carries his own specific set of polygenic variants which are unlikely to be same in another subject with obesity (21).

On the other hand, there are also rare forms of obesity that lack the influence of environmental factors, the so-called recessive monogenic obesity; examples include mutations affecting the leptin gene or its receptor, proopiomelanocortin and pro-hormone convertase subtilisin/kesin type, all leading to a fast and dramatic weight gain (8). These forms present complete penetrance in comparison to the particular form of obesity related to MC4R, and probably MC3R as well; polymorphisms in these genes are associated with forms of obesity that are more severe than typical polygenic obesity but less severe than homozygous gene mutations (8). In contrast to these types of monogenic obesity, the syndromic ones usually occur after infancy, such as Prader-Willi syndrome, Bardet-Biedl syndrome, Albright's hereditary osteodystrophy, Alström syndrome, and WAGR (Wilms' tumor, aniridia, genitourinary anomalies, and retardation) syndrome. Apart from obesity, these syndromes involve dysmorphic features, cognitive impairment, and malformations of major organs (23).

This review summarizes information about the roles of genetic mutations and polymorphisms in the pathogenesis of polygenic obesity in childhood.

## Fat mass and obesity-associated (FTO) gene and obesity

The FTO gene is located on chromosome 16, position 16q12.2, and comprises 9 exons. This gene is expressed in the hypothalamus, at the level of the arcuate nucleus, which is responsible for appetite behavior and fatty acid metabolism, among others (24, 25). Therefore, a recent cross-sectional study was performed on 406 Brazilian children and adolescents aged 7–17 years, among whom 34.5% were overweight or obese. The study proved a positive association between AA genotype of the rs9939609 FTO gene polymorphism and the risk of overweight/obesity (26). Similarly, Cecil et al. stated that the A allele is related to increased fat mass and body mass index (BMI) in Scottish children aged 4–10 years (27). In addition, a study performed on 289 subjects aged 6–19 years proved that the carriers of the AT or AA genotype more frequently expressed a tendency toward intake of food with higher fat content and a loss of food intake control (28). Similarly, a Chinese study performed on children and adolescents found that the AA genotype carriers expressed a preference for a meat-based diet in comparison with those carrying the TT genotype, who preferred a plant-based diet (29). Contradictory results were reported by de Araújo Pereira et al. who did not find any association between FTO gene polymorphisms and overweight or obesity risk in a study performed on 195 obese/overweight individuals with a mean age of 11 years (30). Another study that included 478 African-American children also failed in identifying any association between different FTO gene variants and BMI (31). Therefore, ethnicity might be an important variable regarding the role of FTO gene polymorphism in obesity development. A recent study performed on Polish young adults, which assessed multiple polymorphisms of the FTO gene (rs1121980, rs1421085, rs9930506, and rs9939609), found that this population expressed two disparate haplotypes of the FTO gene variants: TCGA risk haplotype and CTAT protective haplotype, the alleles provided in the previously mentioned order (20). Nevertheless, the genetic susceptibility for obesity can be influenced by dietary interventions in combination with exercise, as stated by Zou et al. in a study performed on obese children carrying the at-risk FTO rs9939609 genotype (32). The rs9939609 FTO gene polymorphism was associated with certain obesity complications, such as high blood pressure, percentage body fat and fat mass, plasma insulin levels, and insulin resistance (33). In contrast, anthropometrical neonatal parameters can influence the nutritional status further on in life. Therefore, a recent study emphasized that a lower PI [i.e., PI, an index computed as birth weight (kg) divided by birth length (m) cubed] suggests that an individual is more vulnerable to the negative effects of the A risk allele of FTO polymorphism on body fat mass (34). In contrast, it was also shown that mothers carrying the variant A allele of rs9939609 FTO polymorphism had newborns with a lower BMI (9). Further functional studies are needed to determine the precise role of the FTO gene in determining obesity and its complications.

## Leptin and obesity

Leptin is a hormone synthesized and secreted into the blood flow by white adipocytes and plays a key role in regulating control of food intake and energy balance. It was proven that, in obese individuals, there is an endogenous leptin resistance mechanism that limits these regulatory effects, explaining the correlation between serum leptin levels and body fat mass (35). This leptin resistance mechanism was related to the presence of the A allele variant of the FTO gene (36), partially explaining the associations of the FTO gene polymorphisms with increased dietary consumption (37) or a hyperphagic phenotype (27, 28). During pregnancy, leptin is synthesized by not only the adipose tissues of both the mother and fetus but also the placenta, and it was proven that the leptin levels in the umbilical cord are positively correlated with birth weight (38, 39). Leptin exerts its roles through the leptin receptor; therefore, the leptin receptor gene is considered a biological pathway related to obesity development (9). A recent study focused on the positive effect of neonatal and maternal leptin gene receptor rs1137101 polymorphism on birth weight and BMI (9). The congenital deficit of leptin results in morbid obesity, severe hyperphagia, hyperinsulinemia or type 2 diabetes mellitus, hypogonadotropic hypogonadism, hypofunction of T cells, and endocrine or metabolic dysfunctions (40, 41). However, mutations in the leptin gene are associated with T cells of normal function and moderate obesity (42). It is well-documented that serum leptin levels depend on age and sex and are correlated with body fat mass (42–44). A study performed on white children assessing 223, 492, and 1019 leptin receptor gene polymorphisms concluded that the most frequent combinations in children with obesity were AG/GG/GA, AG/GG/GG, and AA/GG/GA (10). The same study also underlined that anthropometrical parameters and leptin and adiponectin levels are correlated with the variant genotype of the leptin receptor 223 gene. Therefore, it is well documented that leptin gene receptor 223, 1019, 492, and 976 polymorphisms can modulate the nutritional status in both normal and overweight/obese children (10), but these effects also depend on environmental, nutritional, and social factors. Thus, it was proven that even from birth, weight is positively correlated with maternal fat mass, total body water, body metabolism rate, and metabolic age, whereas it is negatively correlated with maternal smoking status (11). Leptin and leptin gene receptor polymorphisms play a well-established role in childhood obesity.

## Tumor necrosis factor alpha (TNF-α) and obesity

TNF-α is a proinflammatory cytokine expressed as a cell surface transmembrane protein involved in the pathogenesis of multiple inflammatory disorders located on the chromosome 6, site p21.1–21.3. It is also proven to have a catabolic role in infection and cancer but can also be a mediator of cachexia with associated hyperlipidemia (45). Therefore, TNF-α is involved in lipid metabolism leading to hypertriglyceridemia as a result of decreasing lipoprotein lipase activity (46) and increasing the hepatic de novo synthesis of fatty acids (47). It is well known that in subjects with obesity, TNF-α expression is high and correlated with hyperinsulinemia (48). Furthermore, TNF-α was found to regulate leptin expression and secretion (49). A G/A substitution in the promoter region (G-308) of the TNF-α gene was identified (50). Thus, the G-308A polymorphism of this gene is associated with hypertension, leptin levels, and hypercholesterolemia, resulting in metabolic syndrome development (51). In contrast, studies on Caucasian (52) and Chinese (53) populations found correlations between TNF-α 308 G allele and obesity risk. Regarding the presence of different genotypes, Sobti et al. emphasized that the AA and GA genotypes of this gene are more frequently associated with obesity in men, whereas in women, the AG genotype was associated with a higher risk for obesity (54). Nonetheless, a study performed on Iranian subjects failed to prove any correlation between TNF-α 308 G>A gene polymorphism and obesity (55). Similarly, a study performed on Romanian children showed that the variant genotype of TNF-α 308 G>A gene polymorphism was found most frequently in normal weight children (12). Based on all previously mentioned studies, the role of TNF-α 308 G>A gene polymorphism is not clear, but obesity can indeed be considered an inflammatory status.

## Melanocortin 4 receptor (MC4R) and obesity

The MC4R gene is located on chromosome 18q21.32 and, similar to the FTO gene, plays a regulatory role in food intake control and energy balance (56, 57). The common rs17782313 MC4R gene polymorphism was associated with obesity in both European adults and children showing a synergistic effect with FTO gene on obese phenotype (58–60). Rarely, mutations in MC4R gene leading to function loss can lead to monogenic forms of obesity (58), but MC4R-linked obesity is better defined as a particular form which stands between rare recessive monogenic obesity forms and common polygenic ones (8). The rs12970134 and rs17782313 polymorphisms of the MC4R gene were identified to be associated with child and adult obesity, respectively in both Asian and European populations (58, 61–65). A recent study underlined a strong effect of MC4R rs17782313 on body size and fat distribution, proving that the C/C genotype is associated with higher BMI (66). Another study that assessed the same polymorphism of MC4R gene highlighted a significant association with obesity in Mexican children (67). In addition, the authors also identified that both MC4R and FTO risk genotypes increase the metabolic risk in children with obesity (67). The same findings were also identified in Greek children and adolescents with obesity, but the study failed to show an association with the metabolic profile of these children (60). Another important recent discovery is that adiponectin may be involved in mediating the effect of MC4R gene on obesity, based on the findings of Wu et al. who reported that the rs17782313 MC4R gene polymorphism is associated with adiponectin in Chinese children (68). A study performed on newborns in Greece assessed both risk FTO and MC4R variants and concluded that approximately 80% of the Greek population are genetically predisposed to obesity development further on in life (69). According to the results of several studies, the individuals at high risk for obesity development may be homozygous for both FTO and MC4R genes or may be homozygous for one of the two genes and heterozygous for the other (70–72). In contrast, it has also been proven that the rs17782313 MC4R polymorphism has a key role in the eating behavior and control of the eating behavior. Therefore, a recent study performed on children with obesity showed that obese girls that carry the C allele of this genotype express lower satiety responsiveness and higher uncontrolled eating scores in comparison to noncarriers, whereas, in obese boys, the carriers of the same allele present a lower rewarding value of food compared with those who do not carry the C allele Similarly, other studies performed on both adults and children showed that the expression of MC4R and FTO genes can be modulated by lifestyle and physical activity (73, 74).

## Ectoenzyme nucleotide pyrophosphate phosphodiesterase 1 (ENPP1) and obesity

ENPP1 or plasma cell glycoprotein 1 (PC-1) is located on chromosome 6q23.2 and was identified to encode a protein that inhibits insulin signaling (75). The role of this gene in energy metabolism and fat tissue physiology has been widely assessed in the literature (76). K121Q polymorphism was associated with insulin resistance, type 2 diabetes mellitus, and obesity, but the results are contradictory (75, 77–81). In addition to this polymorphism, Bockenski et al. also identified the rs997509 ENPP1 gene polymorphism as a potential contributor to the development of type 2 diabetes mellitus among individuals with obesity (82). Similarly, a study performed on children with obesity underlined the potential role of K121Q ENPP1 polymorphism in not only early perturbations of glucose and insulin metabolism but also subsequent obesity development (83). A large study performed on 1,685 obese and normal-weight Mexican children found nominal associations between different gene polymorphisms, like those in ENPP1 rs7754561 and MC4R rs17782313, and obesity risk or BMI (84). Interestingly, the same authors identified a different pattern for the risk allele of ENPP1 rs7754561 in Mexican individuals showing a protective role in obesity development compared with that in European individuals where the same gene presented positive associations with obesity risk (84). It has also been proven that the overexpression of ENPP1 in human adipocyte cell lines leads to impaired adipocyte maturation (85). In addition to insulin resistance, the K121Q polymorphism was also proven to be associated with different obesity-related phenotypes such as percentage of body fat, fat mass, and plasma insulin levels (86).

## Other genes and obesity

The peroxisome proliferator-activated receptor gamma (PPARG), located on chromosome 3p25.2, plays a role in regulating adipocyte differentiation and influences BMI and glucose metabolism (87). The rs1801282 Pro12Ala polymorphism of this gene has been associated with obesity risk, insulin resistance, type 2 diabetes mellitus, and cardiovascular events (88, 89). A recent study confirmed the association between PPARG and obesity in young adults (66). In addition, a study performed on mothers and their newborns emphasized that homozygous CC carriers of the PPARG genotype presented a higher risk for obesity than heterozygous newborns (13). Moreover, the same authors showed that mothers carrying the homozygous CC genotype of the same gene gave birth to newborns with increased risk for obesity (13).

Angiotensin-converting enzyme (ACE) located on chromosome 17q23.3 has also been shown to have a potential role in obesity development. The ACE rs4646994 has been associated with adiposity and metabolic disorders (90, 91). A potential functional correlation between the PPARG and ACE genes has been underlined based on the fact that PPARG controls the renin-angiotensin system through the transcriptional modulation of renin, angiotensinogen, ACE, and angiotensin II receptor 1 (92). Nevertheless, the results reported in the literature remain contradictory. Certain studies performed on children found that I/I carriers of the ACE gene are associated with higher BMI,^14^ whereas others reported that the D/D genotype of the same gene is linked with increased values of the anthropometric parameters (91, 93). Moreover, other findings have failed to identify any association between ACE gene polymorphisms and body composition (66).

Another class of gene associated with a potential role in obesity development is the glutathione S-transferase (GST), which is involved in different intermediary chemical reactions with glutathione (94). It was proven that the null genotype of both the M1 and T1 alleles of the GST gene (GSTM1 and GSTT1) plays a major protective cellular role against xenobiotic toxic substances and oxidative stress (95, 96). Multiple studies focused on the correlations between these genotypes and neonatal birth weight (15, 97–102).

Interleukin (IL) 6 is a proinflammatory cytokine found to modulate the function of adipose tissue by regulating energy balance, presenting high levels in both obesity and cardiovascular diseases (103–106). Certain genetic studies proved that different polymorphisms of the IL-6 gene present a key role in transcriptional regulation and influence plasmatic cytokine levels, supporting the fact that this gene interacts with metabolic modulation, resulting in different metabolic conditions, such as obesity (107). The G alleles of both IL-6 174 and IL-6 572 genes were associated with obesity and type 2 diabetes mellitus (108). Similarly, in a study performed on Caucasian children, the IL-6572CC, IL-6 190 CC, and IL-6 174 CG genotypes were encountered more frequently in children with obesity, whereas the IL-6 174 CC genotype was found to be a protective factor for childhood obesity (16). Nevertheless, further studies are needed to fully understand the role of the genome in childhood obesity onset.

## Conclusions

Even though recent genetic studies identified over 50 genetic loci, dietary interventions and proper lifestyle changes can prevent obesity development in genetically predisposed people. Therefore, screening programs could be useful in identifying high-risk children, who could benefit from proper prophylactic measures. Perhaps, further studies should also focus on developing targeted genetic therapies designed for children that carry the burden of obesity risk.

## Author contributions

COM, CM, and LEM conceptualized and designed the study, drafted the initial manuscript, and reviewed and revised the manuscript. All authors approved the final manuscript as submitted and agree to be accountable for all aspects of the work.

### Conflict of interest statement

The authors declare that the research was conducted in the absence of any commercial or financial relationships that could be construed as a potential conflict of interest. The reviewer SM and handling editor declared their shared affiliation.

## Abbreviations

ACE: angiotensin-converting enzyme
ENPP1: ectoenzyme nucleotide pyrophosphate phosphodiesterase 1
FTO, fat mass and obesity-associated gene, GST: glutathione S-transferase
IL: interleukin
MC4R: melanocortin 4 receptor gene
PC-1: plasma cell glycoprotein 1
PI: ponderal index
PPARG: peroxisome proliferator-activated receptor gamma
TNF-α: tumor necrosis factor alpha.
